# Single crossover-mediated targeted nucleotide substitution and knock-in strategies with CRISPR/Cas9 system in the rice blast fungus

**DOI:** 10.1038/s41598-019-43913-0

**Published:** 2019-05-15

**Authors:** Tohru Yamato, Ai Handa, Takayuki Arazoe, Misa Kuroki, Akihito Nozaka, Takashi Kamakura, Shuichi Ohsato, Tsutomu Arie, Shigeru Kuwata

**Affiliations:** 1 Graduate School of Agriculture, Meiji University, 1-1-1 Higashi-Mita, Tama-ku, Kawasaki, Kanagawa 214-8571 Japan; 2 Faculty of Science and Technology, Tokyo University of Science, 2641 Yamazaki, Noda, Chiba 278-8510 Japan; 3 Faculty of Agriculture, Tokyo University of Agriculture and Technology, 3-5-8 Saiwai-cho, Fuchu, Tokyo 183-0509 Japan

**Keywords:** Gene expression profiling, Fungal genetics

## Abstract

Clustered regularly interspaced short palindromic repeats (CRISPR)/CRISPR-associated protein 9 (Cas9)-mediated genome editing has become a promising approach for efficient and versatile genetic engineering in various organisms; however, simple and precise nucleotide modification methods in filamentous fungi have been restricted to double crossover type homologous recombination (HR). In this study, we developed a novel genome editing strategy via single crossover-mediated HR in the model filamentous fungus *Pyricularia* (*Magnaporthe*) *oryzae*. This method includes the CRISPR/Cas9 system and a donor vector harboring a single homology arm with point mutations at the CRISPR/Cas9 cleavage site. Using this strategy, we demonstrated highly efficient and freely programmable base substitutions within the desired genomic locus, and target gene disrupted mutants were also obtained via a shortened (100–1000 bp) single homology arm. We further demonstrated that this method allowed a one-step *GFP* gene knock-in at the C-terminus of the targeted gene. Since the genomic recombination does not require an intact protospacer-adjacent motif within the donor construct and any additional modifications of host components, this method can be used in various filamentous fungi for CRISPR/Cas9-based basic and applied biological analyses.

## Introduction

Clustered regularly interspaced short palindromic repeats (CRISPR)/CRISPR-associated protein (Cas) systems derived from prokaryotic adaptive immune system have emerged as RNA-guided revolutionary genome editing technologies^1^. In particular, the type II CRISPR/Cas9 system from *Streptococcus pyogenes* is considerably simpler and widely used as a genome editing tool in several organisms^2–4^. This system comprises only two components: Cas9 endonuclease and single-guide (sg) RNA that is comprised of a CRISPR RNA (crRNA) and trans-activating crRNA (tracrRNA)^4,5^. The sgRNA/Cas9 ribonucleoprotein (RNP) complex searches and binds to the targeted genomic locus by hybridizing the sgRNA/target DNA, and catalyzes a site-specific DNA double-strand break (DSB). The effective binding and cleavage of the *S*. *pyogenes* Cas9 RNP complex requires 5′-NGG-3′ motif, known as protospacer-adjacent motif (PAM), that follows the 3′ end of the complementary target DNA sequence (Fig. 2a).

A majority of the genome editing strategies rely on the induction of DSBs at the specific genomic locus in order to trigger the host DNA repair pathways. In eukaryotes, DSBs are often repaired by two competing pathways, nonhomologous end joining (NHEJ) repair and homologous recombination (HR) repair. In the error-prone NHEJ repair, small insertions and/or deletions occur during the repair of excessive DSBs. This strategy efficiently leads to functional gene disruptions with a frame shift mutation; however, this approach cannot introduce desired mutations. Alternatively, HR-mediated targeted gene modifications can induce more precise editing by adding homologous DNA templates; however, the construction of a target vector is often laborious, and its efficiency depends on the host and its DNA repair property. In addition to these two repair pathways, microhomology-mediated end joining (MMEJ) repair has also been used for targeted gene disruptions and reporter gene knock-ins^6^. This strategy enabled efficient and precise targeted gene disruption and knock-in with short homology arms; however, the desired alteration of the nucleotides cannot be performed.

**Figure 1:**
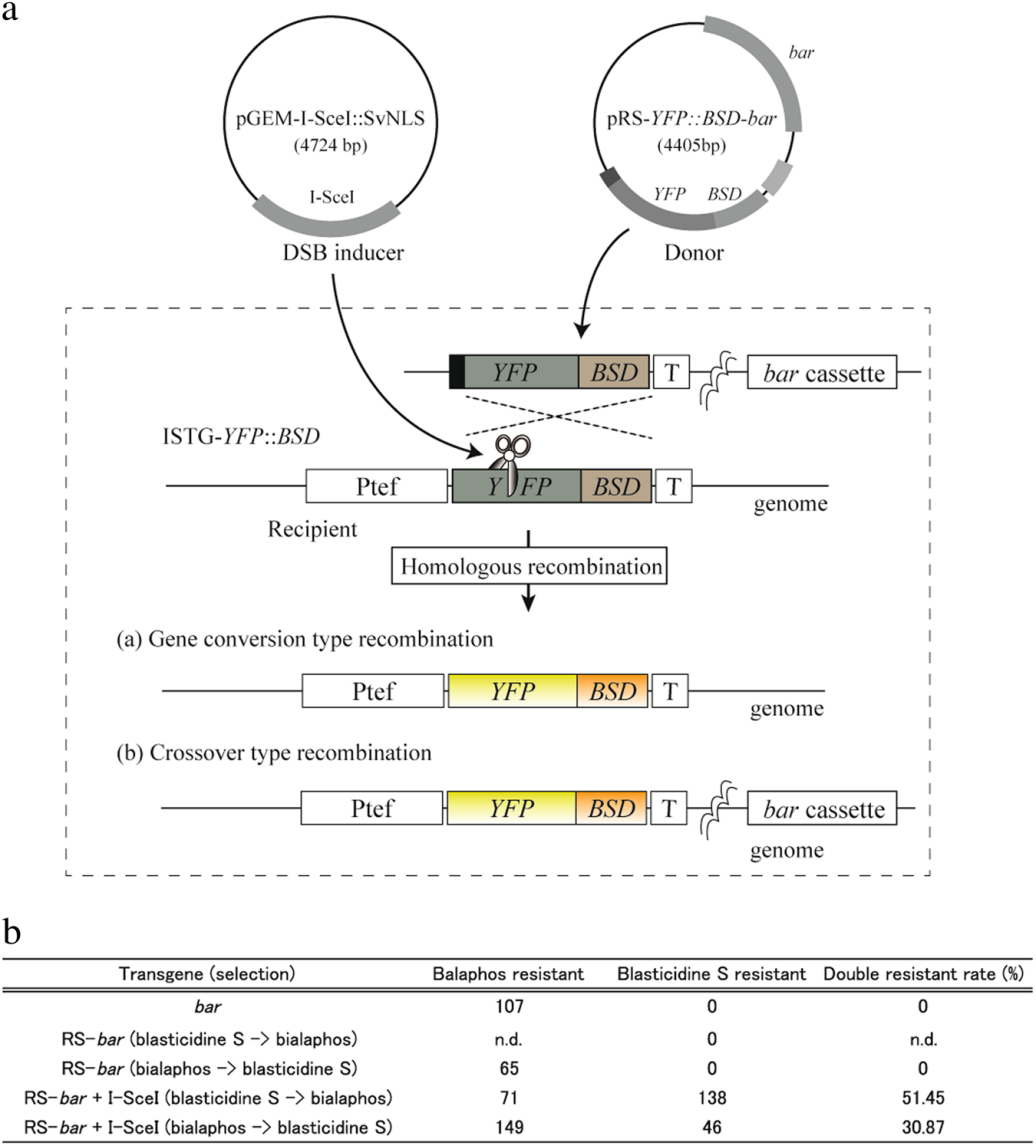
Validation of the single crossover type recombination using the modified homologous recombination (HR) detection/selection system in *Pyricularia oryzae*. (**a**) Schematic representation of the detection and selection strategy for the single crossover type HR. pRS-*YFP::BSD*-*bar* was constructed from pRS-*YFP::BSD* by inserting the bialaphos resistant gene cassette (*bar*). (**b**) The rates of bialaphos-, blastecidine S-, and their double-resistant colonies. N.D.: not determined.

Filamentous fungi comprise a large and diverse group of eukaryotic organisms and are associated with various food, agricultural, and pharmaceutical industries. In contrast, this group contains human and plant pathogens including strains that can produce toxic substances, such as aflatoxin^7^. Therefore, developments in genetic engineering of filamentous fungi can directly affect various fields of basic and applied biological research. CRISPR/Cas9 technologies have been established as one of the simplest genome editing tools in several filamentous fungi^8^ and are still being used in the non-model fungal strains^9–12^. In contrast to other eukaryotes, in a few filamentous fungi, the introduction of DSBs without homologous templates tends to induce large deletions^13–15^; whereas, HR-mediated gene modifications including the MMEJ-mediated knock-in have been demonstrated in most filamentous fungi with higher efficiency than that of the other eukaryotes, excluding yeast^8,15^. The HR repair can be further divided into two sub-pathways, noncrossover (gene conversion) and crossover type recombination^16^. In the gene conversion process, the cleaved DNA site searches homologous or identical sequences as a donor template, and copies their sequence information (Fig. 1a,b). In the crossover process, the DNA strand exchanges occur between the cleaved DNA and donor DNA strands through the resolution of a double holiday junction by a cut at the crossover sites via structure-selective nucleases (Fig. 1a,b)^17,18^. Although crossover type repairs seem to be limited in mitotic DSB repair in mouse embryonic stem cells and human lymphoblastoid cells^19,20^, this has not been validated in the filamentous fungi.

*Pyricularia oryzae* (*Magnaporthe oryzae*) is a model filamentous fungus, which causes destructive fungal diseases in rice. This fungus comprises mononuclear haploid cells and has been established as a basic tool for the analysis of DSB repair and genome editing^13,21–23^. In this study, we used HR detection/selection system to survey whether the crossover type HR has a potential to be applied for the genome editing in *P*. *oryzae*. Using the obtained data, we established simplified and free genome editing strategies for targeted gene disruption, base substitution, and knock-in in the filamentous fungus.

## Results

### Detection and validation of the crossover type HR in the genome of *P*. *oryzae* using reporter gene constructs

We previously established an HR detection/selection system for evaluating the DSB-mediated HR in *P*. *oryzae*^21^. This system comprises two nonfunctional *yellow fluorescent protein* (*YFP*) and *blasticidin S* (BS) *deaminase* (*BSD*) fusion genes (ISTG-*YFP::BSD* and RS-*YFP::BSD*) and a rare-cutting endonuclease I-SceI. To validate whether the crossover type HR can be induced by targeted DSBs in the genome of *P*. *oryzae*, we further developed this system by modifying pRS-*YFP::BSD*, for detecting the crossover type HR. The *bialaphos resistant* (*bar*) gene cassette was inserted into the pRS-*YFP::BSD* vector, which resulted in pRS-*YFP::BSD*-*bar* (donor vector) (Fig. 1a). The donor and I-SceI expression (DSB inducer) vectors were simultaneously introduced into the protoplasts of *P*. *oryzae* transformant ISTG6 that has a single copy of ISTG-*YFP::BSD* (recipient gene) in the genome^22^. Since the recipient gene contains an 18-bp I-SceI recognition site between 327 and 328 bp of the *YFP::BSD* open reading frame, co-introduction with the DSB inducer and donor vector can induce the recipient gene specific DSB and HR between the donor and recipient *YFP::BSD* gene regions (Fig. 1a). The HR occurred-cells would include functionally restored *YFP::BSD* gene. When the gene conversion type HR occurred between the donor and recipient genes, the cells would reveal only blasticidin S resistance. In contrast, the crossover type HR would induce the integration of the whole donor vector sequence into the genome, which would result in double-resistant colonies for BS and bialaphos (Fig. 1a). The transformed protoplasts were selected by BS, and then the obtained colonies were counted and transferred onto PSA medium containing bialaphos (30 µg/mL). Consistent with the previous study, co-introduction with the DSB inducer and donor template generated BS resistant colonies; however, no drug-resistant colonies were obtained when only the donor vector was introduced into protoplasts (Fig. 1b). About a half of the BS resistant colonies showed resistance against bialaphos (Fig. 1b). PCR and sequencing analysis showed that randomly selected double drug resistant colonies (5/5) were produced by crossover type HR. Similar results were observed when the protoplasts were initially selected by bialaphos, and then the colonies were transferred onto the PSA plates with BS. Nevertheless, the double-resistant colonies were fewer than those initially selected by BS (Fig. 1b), suggesting that the initially selected bialaphos resistant colonies comprised the transformants produced by the ectopic insertion of *bar* cassette. These results indicated that introducing site-specific DSB could efficiently induce crossover type HR at the recipient gene locus in *P*. *oryzae*.

### Single crossover-mediated targeted nucleotide substitution with CRISPR/Cas9 system

**Figure 2:**
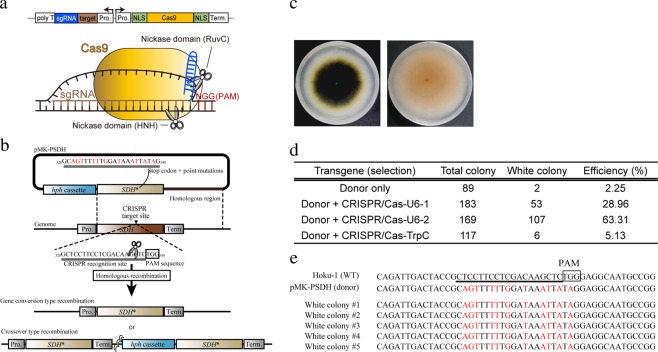
Single crossover-mediated targeted nucleotide substitution of the scytalone dehydratase gene (*SDH*) with CRISPR/Cas9. (**a**) Schematic representation of pCRISPR/Cas vector and its products for the target DNA cleavage. The expressed Cas9 and single-guide (sg) RNA from the pCRISPR/Cas vector form ribonucleoprotein (RNP) complex in the fungal cell. The RNP cleaves the sgRNA/DNA hybrid sequence by catalyzing nickase domain, RuvC, and HNH. The effective cleavage needs the correct protospacer-adjacent motif (PAM: NGG) that follows the target sequence in the complementary DNA strand. Pro.: promoter, Term.: terminator, NLS: nuclear localization signal. (**b**) Schematic representation of the single crossover-mediated *SDH* disruption. The CRISPR/Cas9 target sequence of *SDH* homologous region in pMK-PSDH was modified to evade the CRISPR/Cas9 cleavage and was introduced with a stop codon. *hph*: hygromycin B phosphotransferase. (**c**) Melanin depositions in the wild type (left) and *SDH* disrupted transformants (white colony) (right). (**d**) The efficiencies of the single crossover-mediated *SDH* gene disruption. Total colony: number of hygromycin B-resistant colonies obtained from triplicate experiments. White colony: number of hygromycin B-resistant colonies presenting the white phenotype. Efficiency: percentage of total white colonies. (**e**) Sequences of the CRISPR/Cas9 target region in the wild type, donor vector, and white colony of the transformants.

We previously succeeded in increasing the HR-mediated targeted gene replacement at the *scytalone dehydratase* (*SDH*) gene (MGG_05059) locus via cotransfromation with the fungal CRISPR/Cas9 and targeting vectors^23^. The *SDH* gene disrupted mutants can be detected by the loss of melanin deposition (white phenotype). To induce and evaluate the single crossover-mediated target base substitutions, we constructed a donor vector optimized for the induction of single crossover type HR. The 1335-bp *SDH* region containing point mutations at the CRISPR/Cas9 target site was obtained by fusion PCR, and was cloned into the vector harboring the *hygromycin B phosphotransferase* (*hph*) gene cassette (pMK-PSDH). Because the DNA strand exchanges occur after the DSB repair of cleaved DNA by the copy of the donor DNA sequence, specific DSB-mediated single crossover would induce a pin-point base substitution (stop codon insertion) at the desired *SDH* locus and integrate whole vector sequence containing *hph* gene cassette into the genome (Fig. 2b). In contrast, the gene conversion type HR would occur only in the base substitutions (Fig. 2b). The donor vector and each pCRISPR/Cas-U6-1 site2, pCRISPR/Cas-U6-2 site2, or pCRISPR/Cas-TrpC site2, targeting *SDH* gene^23^, were simultaneously introduced into the wild-type protoplasts and we obtained the hygromycin B resistant colonies. By introducing with the donor and CRISPR/Cas9 vector, the white colony count was dramatically increased, compared with that of only the donor vector (Fig. 2c,d). Sequencing analysis showed that all the white colonies (5/5) comprised desired point mutations (Fig. 2e) and the whole vector sequences were integrated into the *SDH* locus. Consistent with the previous study^23^, in these white colonies, the Cas9 gene was not detected by the PCR analysis using a specific primer set (Table S1). These results indicated that a single crossover-mediated HR was induced by CRISPR-based DSB and this strategy enables targeted base substitutions with high efficiency.

**Figure 3:**
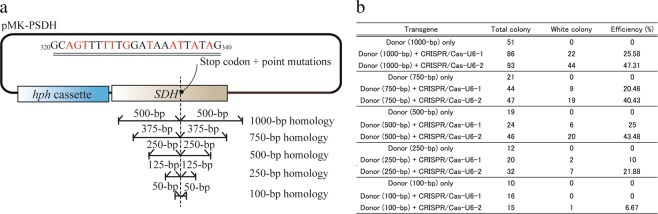
*SDH* disruption using short homology sequences with CRISPR/Cas9. (**a**) Schematic representation of the donor vector having a short homology sequence. (**b**) The efficiencies of *SDH* disruption using the donor vector having a short homology sequence. Total colony: number of hygromycin B-resistant colonies obtained from triplicate experiments. White colony: number of hygromycin B-resistant colonies presenting the white phenotype. Efficiency: percentage of total white colonies.

To assess the feasibility of this strategy, the donor vectors harboring very short homologous sequence (1000, 750, 500, 250, or 100 bp) with stop codon was constructed (Fig. 3a). Both donor and CRISPR/Cas9 vectors were co-introduced into the wild-type protoplasts, and the efficacies of the *SDH* disruption were evaluated by the depleting melanin deposition. White colonies were obtained with high efficiencies (10–25.58%) by co-introduction with pCRISPR/Cas-U6-1 site2 and each donor vector comprising 250–1000 bp homologous sequences (Fig. 3b). The pCRISPR/Cas9-U6-2 could produce more white colonies than pCRISPR/Cas9-U6-1, and it generated the mutant using only 100-bp homologous sequence (Fig. 3b). These results indicated that a single crossover-mediated gene disruption can be performed by using the shortened homology arms in *P*. *oryzae*.

### Single crossover-mediated reporter gene knock-in with CRISPR/Cas9 system

**Figure 4:**
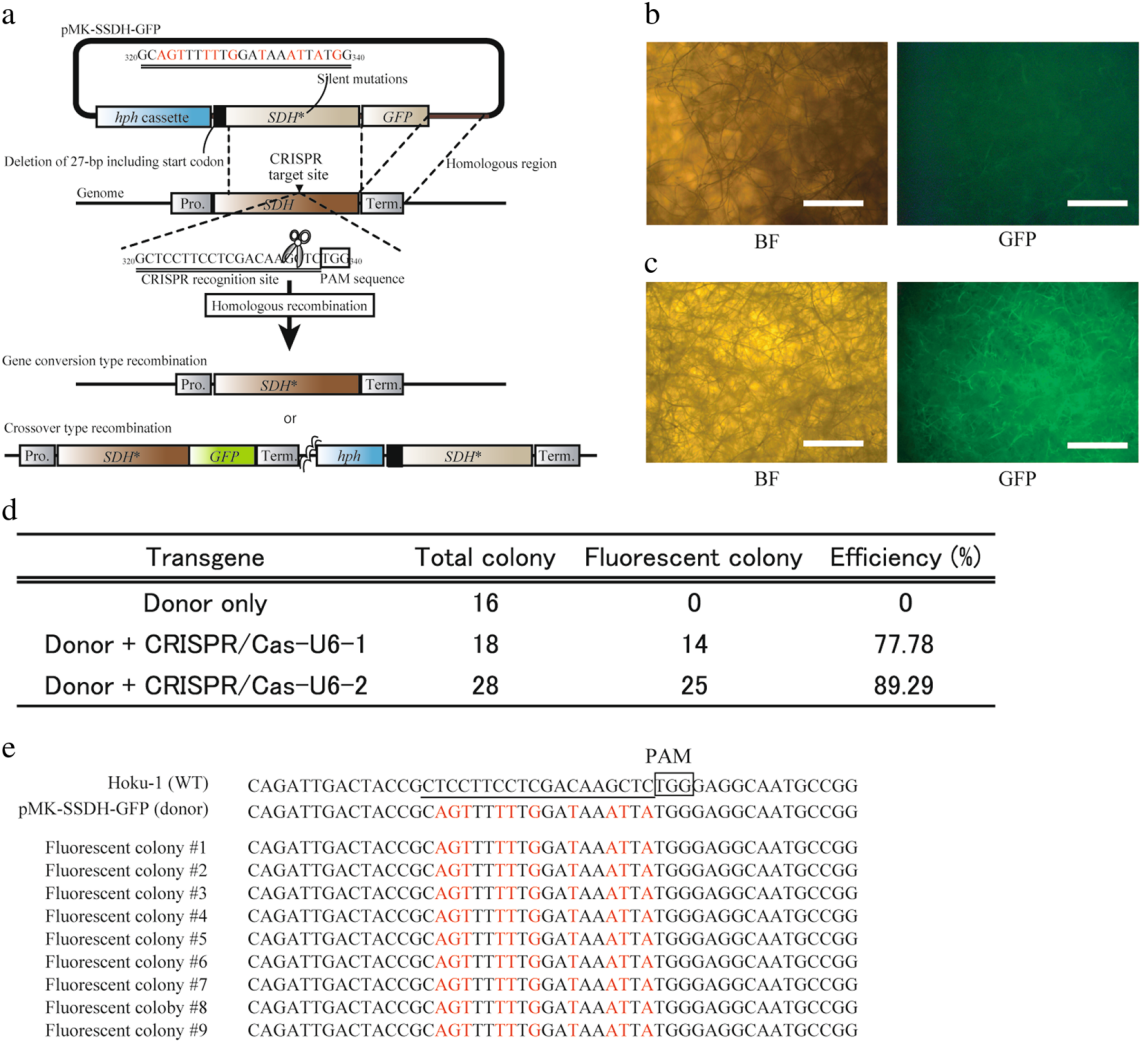
Single crossover-mediated reporter gene knock-in at the *SDH* locus with CRISPR/Cas9. (**a**) Schematic representation of the single crossover-mediated *GFP* knock-in at the *SDH* locus. The start and stop codons were deleted from *SDH* gene and the silent mutations were introduced at the CRISPR/Cas9 target site of this gene. *GFP* was fused to mutated *SDH* C-terminus. (**b**,**c**) GFP fluorescence in the wild type (**b**) and GFP-tagged transformant. (**c**) BF: Bright-field image, GFP: epifluoresence image. The bars are 500 µm. (**d**) The efficiencies of GFP knocked-in transformant. Total colony: number of hygromycin B-resistant colonies obtained from repeated experiments. Fluorescent colony: number of hygromycin B-resistant colonies presenting the GFP fluorescence. Efficiency: percentage of total GFP fluorescent colonies. (**e**) Sequences of the CRISPR/Cas9 target region in the wild type, donor vector, and fluorescent colonies of the transformants.

To investigate whether the single crossover type HR can be applied for the reporter gene knock-in, we constructed a donor vector comprising a *GFP* fused *SDH* homologous sequence. In addition, we introduced silent mutations at the CRISPR/Cas9 target site to avoid the cleavage of the donor vector. Precisely, a single crossover recombination would introduce *GFP* at the C-terminus of the *SDH* gene (Fig. 4a). The hygromycin B resistant colonies with the GFP fluorescence were not obtained via introduction with the only donor vector, whereas co-introduction with the CRISPR/Cas9 and donor vectors helped in obtaining the transformants with the GFP fluorescence (Fig. 4b–d). Randomly selected nine fluorescent colonies comprised the desired silent mutations at the CRISPR/Cas9 target site of the *SDH* gene (Fig. 4e). These results indicated that the CRISPR/Cas9-induced single crossover-type recombination can be applied for the one-step reporter gene knock-in at the targeted genomic locus.

### Functional and expression analysis of *Spo11* using single crossover-mediated gene disruption and reporter gene knock-in

To assess whether the single crossover-mediated genome editing is effective for other genomic loci, we analyzed the functions and expression of *SPO11* (MGG_10666) in *P*. *oryzae*. In *Saccharomyces cerevisiae*, Spo11 catalyzes DSB formation to act via topoisomerase-like reaction in meiosis^24^. Since asexual reproduction is predominantly observed in *P*. *oryzae*, the function of genes related to the meiotic recombination in asexual life cycle has not been characterized. We created two donor vectors for the functional disruption (Fig. S1) and GFP tagging of Spo11 (Fig. S2). When seven independent well-grown colonies obtained via co-introduction with CRISPR/Cas9 and donor vectors for functional disruption (pMK-PSPO) were subjected to sequencing analysis, three out of seven transformants had desired stop codons at the *SPO11* gene (ca. 43%); however, the three mutants indicated no significant differences in the growth, conidiation, germination, and appressorium formation rates, compared with those of the wild-type strain. For the Spo11-GFP plus CRISPR/Cas9 co-transformation, ten hygromycin B-resistant colonies were randomly selected and two (20%) contained the desired allelic replacement based on PCR and sequencing analysis; however, the GFP fluorescence was not observed during the vegetative growth and appressorium formation. These results suggest that the single-crossover strategy could be applied universally across genomic loci. Furthermore, we have employed this strategy to functionally demonstrate that Spo11 does not play a role in the asexual life cycle of *P*. *oryzae*.

## Discussion

We demonstrated that a single crossover type HR could be induced by the site-specific DSB, which allows free and efficient genome editing including targeted nucleotide substitutions and reporter gene knock-ins in the model filamentous fungus *P*. *oryzae*. Because the CRISPR/Cas9 system derived from *S*. *pyogenes* is considerably simpler than the other CRISPR/Cas systems, this strategy would be useful for various types of research and can be applied to a majority of filamentous fungi. To the best our knowledge, this is the first demonstration of efficient and desired base editing without additional modifications of host components, such as NHEJ related genes disruptions, in filamentous fungi.

The easiest functional gene disruption method with the CRISPR/Cas system is the NHEJ-mediated mutagenesis because this strategy only needs to integrate the CRISPR/Cas system into the genome. Nevertheless, in filamentous fungi, the repair of DSBs without donor templates often induces undesired large deletions or mutations^13–15^. Additionally, the constitutive expression of the CRISPR/Cas9 system would trigger mutations outside of the target site (off-target effect), which might affect the phenotypes of interest^25^. Using the CRISPR/Cas9 system and the other nucleases, HR could be efficiently induced via the transient expression of the nucleases in fungal protoplasts^8^, which would allow the minimization of the off-target effects. The single crossover-mediated genome editing is easier than the double crossover-mediated gene replacement because the donor vector can be constructed via one-step cloning. Moreover, as the single crossover-mediated recombination enables the integration of the whole plasmid structure into the targeted genomic locus via DNA strand exchange, a large genomic region, such as a metabolic gene cluster, could be efficiently knocked-in at the desired locus.

The most significant limitation of the CRISPR/Cas system is the requirement of the PAM sequence for effective DNA cleavage. Recently, various CRISPR/Cas homologs and orthologs, with various types of the PAM, have been identified from a broad range of bacteria and archaea, and their application in flexible genome editing tools is progressing^26^. The protein engineering of Cas9 also enabled modifications in the PAM recognition^27^. Nevertheless, for pin-point genome and base editing, it is necessary to use the appropriate CRISPR/Cas homolog, ortholog, or engineered Cas protein every time. One of the advantages of our strategy is that its target bases can be freely modified independent of the PAM sequence. We anticipate that the single crossover-mediated genome editing strategy can expand the scope of the genome editing technology, and aid a variety of basic and applied biological research in filamentous fungi.

## Methods

### Fungal strain, growth conditions, and DNA analysis

The *P*. *oryzae* strain Hoku-1 was used as a wild-type strain in this study. The media composition and genomic DNA extraction were as described previously^21^. PCR and sequencing analysis were performed via standard procedures^22^. BLAST search was performed using http://blast.be-md.ncbi.nlm.nih.gov/Blast.cgi. Primers and oligonucleotides used in this study are listed in Table S1.

### Construction of a single crossover detection/selection system and its validation

For detecting the single crossover events in the genome of *P*. *oryzae*, the *YFP::BSD*-based HR detection/selection system^22^ was modified. To construct pRS-*YFP::BSD*-*bar*, as a donor vector for the single crossover, the *bar* gene cassette was excited from pGEM-*bar*^22^ via ApaI digestion and was inserted into the ApaI site of pRS-*YFP::BSD*^22^ which resulted in pRS-*YFP::BSD*-*bar*. The pGEM-I-SceI::SvNLS and ISTG6 strains were used as DSB inducers and recipient host cells, respectively^22^. The preparation of ISTG protoplasts and PEG transformation were described previously^22^, and the procedures were slightly modified. Briefly, 2.5 µg of each pRS-*YFP::BSD-bar* and pGEM-I-SceI::SvNLS vectors was mixed and added to the protoplasts. About 24 h after transformation, the protoplasts were transferred onto a PSA plate with BS (200 µg/mL) or bialaphos sodium (30 µg/mL). For the validation of single crossover-mediated HR between the ISTG region and pRS-*YFP::BSD-bar*, the PCR analysis was performed by using a YFP-seq-1/TTrpC-1 primer set. The PCR products were sequenced by using the YFP-seq-1.

### Construction of CRISPR/Cas9 expression vector targeting endogenous genes

Sense (SPO-gRNA-1) and antisense (SPO-gRNA-2) oligonucleotides for the CRISPR/Cas9 targeting *SPO11* were designed using the website service, CRISPR direct (http://crispr.dbcls.jp), and were annealed according to the procedures of the previous report^28^ (Table S1). The annealed oligonucleotides were inserted into pCRISPR/Cas-U6-2 via the Golden Gate cloning method according to the previous report^23^ with certain modifications. Briefly, 0.8 µL 50-fold-diluted oligonucleotides, 0.3 µL pCRISPR/Cas-U6-2, 0.2 µL Esp3I enzyme (Thermo Scientific), 0.2 µL Quick ligase (New England Biolabs), and 0.4 µL 10 × T4 DNA ligase buffer (New England Biolabs) were mixed in a single tube and the volume was made up to 5 µL by adding sterilized water. The mixtures were then subjected to a thermal cycling reaction as follows: 6 cycles of 37 °C for 5 min and 16 °C for 10 min. After the cycling reaction, 1 µL of the reaction mixtures was used for transformation of *Escherichia coli* DH5α chemical competent cells.

### Construction of donor vectors for single crossover recombination

The *SDH* gene region comprising point mutations was amplified via fusion PCR methods. For the first PCR, the *SDH* gene regions were amplified from the genome of *P*. *oryzae* using a PSDH-1/-2 primer set and PSDH-3/-4 set, respectively. The PCR products obtained from the first PCR were mixed and used for the second PCR. The fusion PCR product was generated by a PSDH-1/-4 primer set and cloned between the HindIII and SpeI sites of pMK412-dGFP^21^, which resulted in pMK-PSDH. The donor vectors with short homology regions (pMK-PSDH1000, pMK-PSDH750, pMK-PSDH500, pMK-PSDH250, and pMK-PSDH100) were constructed from pMK-PSDH via PCR-based methods and cloned between HindIII and SpeI sites of pMK412-dGFP. The PSDH-1/PSDH1000-2, PSDH-1/PSDH750-2, PSDH500-1/-2, PSDH250-1/-2, and PSDH100-1/-2 were used for the amplification of 1000, 750, 500, 250, and 100 bp of *SDH* region with mutations, respectively. The donor vector for GFP knock-in at the C-terminus of the *SDH* gene was constructed via fusion PCR methods. For the first PCR, the *SDH* gene regions with silent mutations, which removed the start and stop codons, were amplified from pMK-PSDH using a KSDH-1/-2 primer set and KSDH-3/-4 primer set, respectively. The PCR products obtained from the first PCR were mixed and used for the second PCR. The fusion PCR product was generated by a KSDH-1/-4 primer set. The *GFP* gene and putative terminator region of the *SDH* gene were also amplified from pMK412^21^ and pMK-PSDH using KGFP-1/-2 and KSDH-5/PSDH-4 primer sets, respectively. The PCR products were mixed and used for second PCR, and the fusion PCR product was generated by a KGFP-1/PSDH-4 primer set. The fusion PCR products generated by the KSDH-1/-4 and KGFP-1/KSDH-6 primer sets were mixed and used for the third PCR. The third PCR product was generated by a KSDH-1/PSDH-4 primer set and cloned between the HindIII and SpeI sites of pMK-dGFP, which resulted in pMK-KSDH-GFP. The *SPO11* gene region with point mutations was constructed via fusion PCR methods. For the first PCR, the *SPO11* gene regions were amplified from the genome of *P*. *oryzae* using PSPO-1/-2 and PSPO-3/4 primer sets, respectively. The PCR products obtained from the first PCR were mixed and used for the second PCR. The fusion PCR product was generated by a PSPO-1/-4 primer set and was cloned between the HindIII and SpeI sites of pMK412-dGFP, which resulted in pMK412-PSPO. The donor vector for GFP knock-in at the C-terminus of the *SPO11* gene was constructed via fusion PCR methods. For the first PCR, the *SPO11* gene regions with silent mutations, which removed the start and stop codons, were amplified from the pMK-KSPO and genome using a KSPO-1/-2 and KSPO-3/-4 primer sets, respectively. The PCR products obtained from the first PCR were mixed and used for the second PCR. The fusion PCR product was generated by a KSPO-1/KSPO-4 primer set and was cloned between the SpeI and SacI sites of pMK412-dGFP, which resulted in pMK-KSPO. The *GFP* gene and terminator region of *SPO11* were amplified from the pMK412 and genome using the KGFP-3/-4 and KSPO-5/PSPO-4 primer sets, respectively. The PCR products obtained from the first PCR were mixed and used for the second PCR. The fusion PCR product was generated by a KGFP-1/PSPO-4 primer set and was cloned between the AscI and PacI sites of pMK-KSPO, which resulted in pMK-KSPO-GFP.

### Transformation of *P*. *oryzae* with CRISPR/Cas9 and donor vectors

The 2.5 µg of each CRISPR/Cas9 vector and donor vector was mixed and added to the protoplasts. About 24 h after transformation, the protoplasts were transferred onto a PSA plate with hygromycin B (200 µg/mL).

## Supplementary information

Supplementary information

## Data Availability

All data generated or analyzed during this study are included in this published article and its Supplementary Information Files, or are available from the corresponding author in reasonable request.
