# Supplementary material for: Single crossover-mediated targeted nucleotide substitution and knock-in strategies with CRISPR/Cas9 system in the rice blast fungus
Source: Sci Rep. 2019 May 15;9:7427. doi: 10.1038/s41598-019-43913-0 (PMC6520371; doi:10.1038/s41598-019-43913-0)
Supplement: Supplementary file 1 — Supplementary information [file 41598_2019_43913_MOESM1_ESM.docx]

**SUPPLEMENTARY INFORMATION**

**Scientific Reports**

**Single crossover-mediated targeted nucleotide substitution and knock-in strategies with CRISPR/Cas9 system in the rice blast fungus**

Tohru Yamato, Ai Handa, Takayuki Arazoe^*^, Misa Kuroki, Akihito Nozaka, Takashi Kamakura, Shuichi Ohsato, Tsutomu Arie, Shigeru Kuwata^*^

^a^Graduate School of Agriculture, Meiji University, 1-1-1 Higashi-Mita, Tama-ku, Kawasaki, Kanagawa 214-8571, Japan

^b^Faculty of Science and Technology, Tokyo University of Science, 2641 Yamazaki, Noda, Chiba 278-8510, Japan

cFaculty of Agriculture, Tokyo University of Agriculture and Technology, 3-5-8 Saiwai-cho, Fuchu, Tokyo 183-0509, Japan

Corresponding author:

Address: Faculty of Science and Technology, Tokyo University of Science, 2641 Yamazaki, Noda, Chiba 278-8510, Japan.

Tel.:+81 47 124 1501 (ext. 3426).

E-mail: arazoe@rs.tus.ac.jp

Graduate School of Agriculture, Meiji University, 1-1-1 Higashi-Mita, Tama-ku, Kawasaki-shi, Kanagawa 214-8571, Japan.

Tel.: +81 44 934 7036.

E-mail: kuwata@meiji.ac.jp


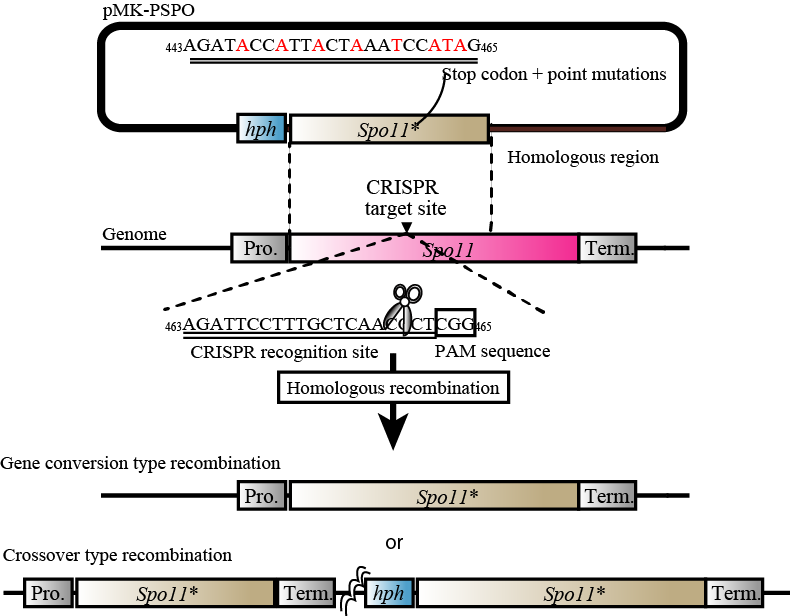


**Figure S1**. Schematic representation of the single crossover-mediated *Spo11* disruption. The CRISPR/Cas9 target sequence of *Spo11* homologous region in pMK-PSPO was modified to evade the CRISPR/Cas9 cleavage and was introduced with stop codon. *hph*: hygromycin B phosphotransferase.


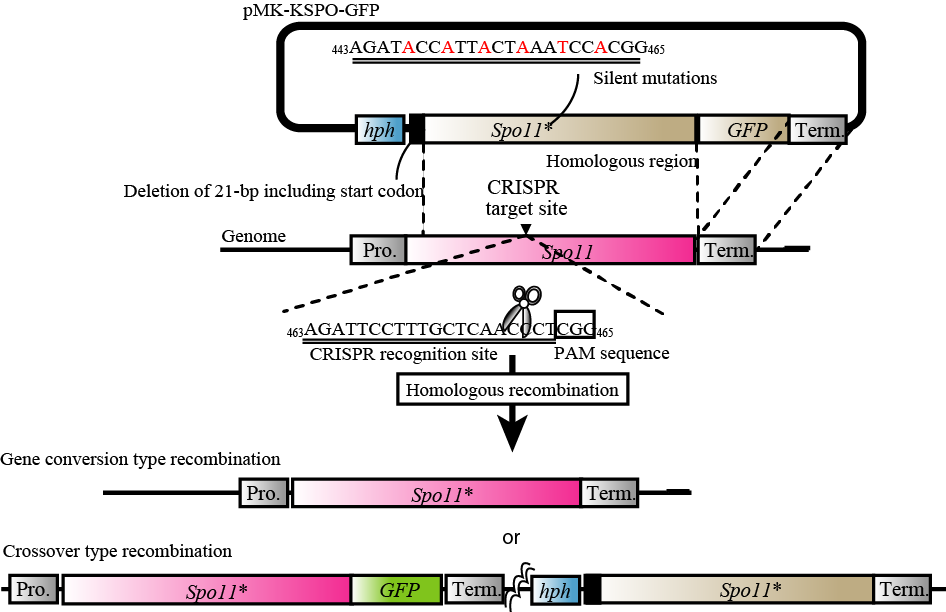


**Figure S2**. Schematic representation of the single crossover-mediated *GFP* knock-in at the *Spo11* locus. The start and stop codons were deleted from the *Spo11* gene and the silent mutations were introduced at the CRISPR/Cas9 target sequence of this gene. *GFP* was fused to the mutated *Spo11* C-terminus. *hph*: hygromycin B phosphotransferase.

**Supplementary Table 1.** List of oligonucleotides used in this study

| Name | Oligonucleotide sequence (5′- 3′) | Experiment |
| --- | --- | --- |
| TTrpC-1 | TTGGGCCCGATCCTCTAGAAAGAAGGATTACCTCTAAA | TrpC terminator targeted oligonucleotide for the detection of single crossover-mediated HR |
| YFP-seq-1 | ATGGTGAGCAAGGGCGAGGAGCTGT | ISTG*-YFP::BSD* target oligonucleotide for the detection of single crossover-mediated HR and sequencing analysis |
| Cas-det-3 | ACTCGACCCGACAAGGCCGAT | Cas9 gene target oligonucleotide for the detection of Cas9 gene integration |
| Cas-det-4 | GGAAGAGATCCGCGTACTGG | Cas9 gene target oligonucleotide for the detection of Cas9 gene integration |
| SPO-gRNA-1 | TTCGAGATTCCTTTGCTCAACCCT | *Spo11* targeted gRNA oligonucleotide for the construction of pCRISPR/Cas9 expression vector |
| SPO-gRNA-2 | AAACAGGGTTGAGCAAAGGAATCT | *Spo11* targeted gRNA oligonucleotide for the construction of pCRISPR/Cas9 expression vector |
| PSDH-1 | cgaaagcttatgggttcgcaagttcaaaag | *SDH* region target oligonucleotide for the construction of pMK-PSDH, -PSDH1000, and -PSDH750 |
| PSDH-2 | CCTATAATTTATCCAAAAAACTGCGGTAGTCAATCTGTTTTTTT | *SDH* region target oligonucleotide for the construction of pMK-PSDH |
| PSDH-3 | TTTTTTGGATAAATTATAGGAGGCAATGCCGGCCGAGGAGTTCG | *SDH* region target oligonucleotide for the construction of pMK-PSDH |
| PSDH-4 | agaactagttctttgtttatacttgctaat | *SDH* region target oligonucleotide for the construction of pMK-PSDH and -KSDH-GFP |
| PSDH1000-2 | agaactagtgcacgtttcgtcgcatctttgacgatttag | *SDH* region target oligonucleotide for the construction of pMK-PSDH1000 |
| PSDH750-2 | AAACTCATGGTGGTGTCCTTGTA | *SDH* region target oligonucleotide for the construction of pMK-PSDH750 |
| PSDH500-1 | AGTTGGACTGGGATAGGCTGCGAA | *SDH* region target oligonucleotide for the construction of pMK-PSDH500 |
| PSDH500-2 | AAACTTCGCAGCCTATCCCAGTCC | *SDH* region target oligonucleotide for the construction of pMK-PSDH500 |
| PSDH250-1 | AGTTGCTCCTTCCTCGACAAGCTC | *SDH* region target oligonucleotide for the construction of pMK-PSDH250 |
| PSDH250-2 | AAACGAGCTTGTCGAGGAAGGAGC | *SDH* region target oligonucleotide for the construction of pMK-PSDH250 |
| PSDH100-1 | AGTTGTCCCGCACCAGAGGTACA | *SDH* region target oligonucleotide for the construction of pMK-PSDH100 |
| PSDH100-2 | AAACTGTACCTCTGGTGCGGGAC | *SDH* region target oligonucleotide for the construction of pMK-PSDH100 |
| KSDH-1 | gataagcttgagataaccttctcaggtgagcata | *SDH* region target oligonucleotide for the construction of pMK-KSDH-GFP |
| KSDH-2 | CCCATAATTTATCCAAAAAACTGCGGTAGTCAATCTGTTTTTTT | *SDH* region target oligonucleotide for the construction of pMK-KSDH-GFP |
| KSDH-3 | TTTTTTGGATAAATTATGGGAGGCAATGCCGGCCGAGGAGTTCG | *SDH* region target oligonucleotide for the construction of pMK-KSDH-GFP |
| KSDH-4 | gctcctcgcccttgctcaccattttgtcgccaaaggtctcccgt | *SDH* region target oligonucleotide for the construction of pMK-KSDH-GFP |
| KGFP-1 | acgggagacctttggcgacaaaatggtgagcaagggcgaggagc | *GFP* gene target oligonucleotide for the construction of pMK-KSDH-GFP |
| KGFP-2 | ccggcacgcatgatgcatgcatttacttgtacagctcgtccatg | *GFP* gene target oligonucleotide for the construction of pMK-KSDH-GFP |
| KSDH-5 | catggacgagctgtacaagtaaatgcatgcatcatgcgtgccgg | *SDH* region target oligonucleotide for the construction of pMK-KSDH-GFP |
| PSPO-1 | gataagcttatgctcaggcactcggagcgtgtccgcagg | *Spo11* region target oligonucleotide for the construction of pMK-PSPO |
| PSPO-2 | CTATGGATTTAGTAATGGTATCTTACCAAACGACCTTGCCTCAG | *Spo11* region target oligonucleotide for the construction of pMK-PSPO |
| PSPO-3 | ACCATTACTAAATCCATAGAGACATAGTACGTTTCTGCGGGTTG | *Spo11* region target oligonucleotide for the construction of pMK-PSPO |
| PSPO-4 | AGAACTAGTAAATAATTGTTCCCTAGAGGGGCATTACAATCAAC | *Spo11* region target oligonucleotide for the construction of pMK-PSPO and sequencing analysis |
| KSPO-1 | GGCGCTAGCGTCCGCAGGCCTTCGAGGCGTCTGCTGTTA | *Spo11* region target oligonucleotide for the construction of pMK-KSPO |
| KSPO-2 | CCGTGGATTTAGTAATGGTATCTTA | *Spo11* region target oligonucleotide for the construction of pMK-KSPO |
| KSPO-3 | ATACCATTACTAAATCCACGGAGACATAGTACGTTTCTGC | *Spo11* region target oligonucleotide for the construction of pMK-KSPO |
| KSPO-4 | ctggagctcttaattaaCGGCGCGCCTGCATTCGTCAACTGGGAGCAAAGCTTTTT | *Spo11* region target oligonucleotide for the construction of pMK-KSPO |
| KGFP-3 | GCAGGCGCGCCGATGGTGAGCAAGGGCGAGGAGCTGT | *GFP* gene target oligonucleotide for the construction of pMK-KSPO-GFP |
| KGFP-4 | GTAGCAAATCCCGTTCTGGTTACTTGTACAGCTCGTCCATGCCG | *GFP* gene target oligonucleotide for the construction of pMK-KSPO-GFP |
| KSPO-5 | GGACGAGCTGTACAAGTAACCAGAACGGGATTTGCTACAGGTCA | Putative *SPO11* terminator target oligonucleotide for the construction of pMK-KSPO-GFP |
| KSPO-6 | CTCTTAATTAACTCTTTCTGTTGGAGGGGTTCTTTACATTT | Putative *SPO11* terminator target oligonucleotide for the construction of pMK-KSPO-GFP |
| SDH-Seq-1 | CGAAACCCCTTTTTGGGGCTAATTAGTAAA | *SDH* region target oligonucleotide for the sequencing analysis |
| SDH-Seq-2 | ATTTATTTGTCGCCAAAGGTCTCCCGTC | *SDH* region target oligonucleotide for the sequencing analysis |
| SPO-Seq-1 | TCTCATTACACTTCCTGACTTGGCA | Spo11 region target oligonucleotide for the sequencing analysis |
